# Five candidate biomarkers associated with the diagnosis and prognosis of cervical cancer

**DOI:** 10.1042/BSR20204394

**Published:** 2021-03-10

**Authors:** Hong-Yan Han, Jiang-Tao Mou, Wen-Ping Jiang, Xiu-Ming Zhai, Kun Deng

**Affiliations:** Department of Laboratory Medicine, The Third Affiliated Hospital of Chongqing Medical University (Gener Hospital), Chongqing 401120, China

**Keywords:** biomarkers, cervical cancer, differentially expressed genes, machine learning

## Abstract

**Purpose:** Cervical cancer (CC) is one of the most general gynecological malignancies and is associated with high morbidity and mortality. We aimed to select candidate genes related to the diagnosis and prognosis of CC.

**Methods:** The mRNA expression profile datasets were downloaded. We also downloaded RNA-sequencing gene expression data and related clinical materials from TCGA, which included 307 CC samples and 3 normal samples. Differentially expressed genes (DEGs) were obtained by R software. GO function analysis and Kyoto Encyclopedia of Genes and Genomes (KEGG) pathway enrichment analysis of DEGs were performed in the DAVID dataset. Using machine learning, the optimal diagnostic mRNA biomarkers for CC were identified. We used qRT-PCR and Human Protein Atlas (HPA) database to exhibit the differences in gene and protein levels of candidate genes.

**Results:** A total of 313 DEGs were screened from the microarray expression profile datasets. DNA methyltransferase 1 (DNMT1), Chromatin Assembly Factor 1, subunit B (CHAF1B), Chromatin Assembly Factor 1, subunit A (CHAF1A), MCM2, CDKN2A were identified as optimal diagnostic mRNA biomarkers for CC. Additionally, the GEPIA database showed that the DNMT1, CHAF1B, CHAF1A, MCM2 and CDKN2A were associated with the poor survival of CC patients. HPA database and qRT-PCR confirmed that these genes were highly expressed in CC tissues.

**Conclusion:** The present study identified five DEmRNAs, including DNMT1, CHAF1B, CHAF1A, MCM2 and Kinetochore-related protein 1 (KNTC1), as potential diagnostic and prognostic biomarkers of CC.

## Introduction

Cervical cancer (CC) is one of the most common gynecological malignant tumors worldwide and has become a prominent public health issue [1,2]. According to reports, the incidence of CC ranks second among female malignant tumors in the world, and the mortality rate ranks first among female malignant tumors of the reproductive system. It is a serious threat to women’s health [3]. CC is difficult to diagnose at the early stage, leading to a delay in effective treatment [4]. At present, surgery, chemotherapy and radiotherapy are the most commonly used treatment methods for CC; however, due to the resistance of CC cells to therapeutic drugs, chemotherapy drugs are relatively ineffective in treating CC [5,6]. Therefore, it is of great significance to develop new diagnostic or treatment methods for CC.

Gene Expression Omnibus (GEO) was launched in response to the growing demand for public repositories of high-throughput gene expression data [7]. The Cancer Genome Atlas (TCGA) project analyzes DNA copy number, mRNA expression, promoter methylation, microRNA expression of 307 cervical SCCs, and 3 normal cervical tissue samples [8,9]. Therefore, combining the GEO and TCGA datasets may provide an important perspective for the study of new biomarkers. In fact, there are many reports that revealed a series of highly specific and sensitive markers by screening for tumor biomarkers based on GEO and TCGA data recently [10]. Compared with conventional screening methods, analyzing high-throughput data based on bioinformatics methods allows researchers to obtain stable and reliable biomarkers in a larger number of clinical samples.

In the present study, we downloaded five original mRNA microarray datasets from the GEO database, GSE7410, GSE7803, GSE9750, GSE55940 and GSE63514, a total of 176 samples containing 68 normal cervical samples and 108 CC specimens. We also downloaded RNA-sequencing gene expression data and related clinical materials from TCGA, which included 307 CC samples and 3 normal samples. The differentially expressed genes (DEGs) in normal tissues and tumor samples were obtained by R software. By evaluating the diagnostic and prognostic value of these DEGs, five DEGs associated with the diagnostic and prognosis of CC were screened out.

## Materials and methods

### Microarray data

Using the keywords ‘cervical cancer’ to search on the GEO database, the raw gene expression profiles of GSE7410, GSE7803, GSE9750, GSE55940 and GSE63514 were downloaded. RNA-sequencing gene expression data and related clinical materials from TCGA were retrieved on 24 March 2020, and comprised data from 307 CC samples and 3 normal samples. The inclusion criteria were pathologically confirmed CC, complete RNA expression data from the patients [11].

### Identification of DEGs and functional enrichment analysis

Data analysis was conducted using package limma in R language (version 3.4.0) [12]. FDR < 0.01 and |Combined.ES| > 1.5 were set up to screen DEGs. Heat maps were generated by pheatmap package in R. Through the DAVID database [13], functional enrichment analysis was conducted to identify gene ontology (GO) annotation and Kyoto Encyclopedia of Genes and Genomes (KEGG). GO terms and KEGG pathways with *P*<0.05 were selected.

### Identification of optimal diagnostic mRNA biomarkers for CC

Modeling and machine learning algorithm were used to identify optimal diagnostic mRNA biomarkers for CC. The procedure was performed as previously described [14]. ROC curve was used to explore the differentially diagnostic capability of the five candidate genes.

### Identification of survival-related DEGs and establishment of the prognostic gene signature

Using the survival and survminer package in R, Kaplan–Meier (KM) plots and log-rank tests were performed to elucidate the relationship between 5-year overall survival (OS) rates and DEGs expression levels. DEGs with *P*<0.01 were considered statistically significant and included in subsequent analyses. For these prognostic genes, LASSO Cox regression analysis was used to determine the gene signature prognostic model using the following formula to calculate the risk score for each sample. $\text{riskScore}=\sum_{\text{i}=1}^{\text{n}}\exp_{\text{i}}\times \beta_{\text{i}}$. Patients with CC were divided into high-risk and low-risk groups based on the median risk score, and KM survival analysis was performed between the low-risk and high-risk groups. ROC curve analysis to evaluate the predictive power of the forecasting model.

### Quantitative RT-PCR confirmation and validation in datasets

According to the results of GEO integrated analysis, we selected six DEGs (DNA methyltransferase 1 (DNMT1), Chromatin Assembly Factor 1, subunit B (CHAF1B), Chromatin Assembly Factor 1, subunit A (CHAF1A), MCM2, CDKN2A, Kinetochore-related protein 1 (KNTC1)) in CC versus normal control as candidate genes. A total of 13 blood samples were collected from 7 normal subjects and 6 patients diagnosed with CC. Informed written consent was obtained from all participants, and research protocols were approved by the Ethics Committee of our hospital.

To evaluate mRNA expression of candidate genes, we used GEO database and TCGA database to differentiate expression of candidate genes in CC tissues and normal tissues.

### Evaluation of immunohistochemical staining

To validate the protein expression level of candidate genes in CC tissues, we used Human Protein Atlas (HPA, https://www.proteinatlas.org/) database to obtain immunohistochemical staining.

### Relationship between candidate genes and clinical features in hepatocellular carcinoma patients

To further explore the relationship between candidate genes and tumor clinical features, we analyzed the TCGA clinical data using LinkedOmics (http://www.linkedomics.org/) database.

## Results

### Identification of DEGs in CC

**Figure 1:**
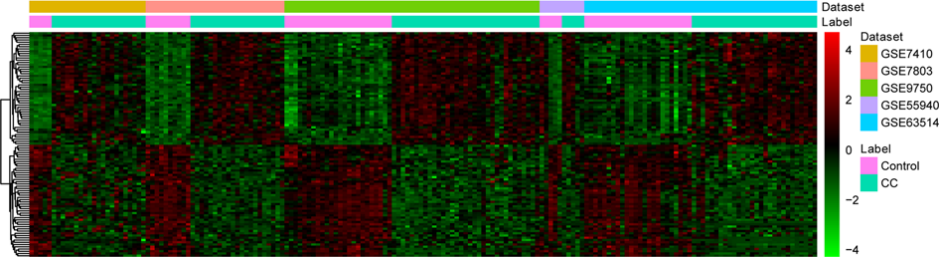
Hierarchical clustering analysis of top 100 DEGs in CC based on GEO (fold change > 2.0, *P*<0.05 in *t* test)

After retrieving, we obtained five microarray datasets of mRNA according to the inclusion criteria from the GEO database. The characteristics of the individual database for the integrated analysis are displayed in Table 1. A total of 313 DEGs were obtained, including 200 up-regulated and 113 down-regulated genes. The cluster heatmap of top 100 DEGs in all samples are shown in Figure 1.

**Table 1:** Details for GEO CC data

GEO ID	Samples (Normal:CC)	Type	Platform	Year	Author	Type
GSE7410	5:21	mRNA	GPL1708	2008	Biewenga	Tissue
GSE7803	10:21	mRNA	GPL96	2007	Zhai	Tissue
GSE9750	24:33	mRNA	GPL96	2008	Murty	Tissue
GSE55940	5:5	mRNA	GPL16238	2014	Ye	Tissue
GSE63514	24:28	mRNA	GPL570	2015	den Boon	Tissue

### GO and KEGG pathway analysis of DEGs

**Figure 2:**
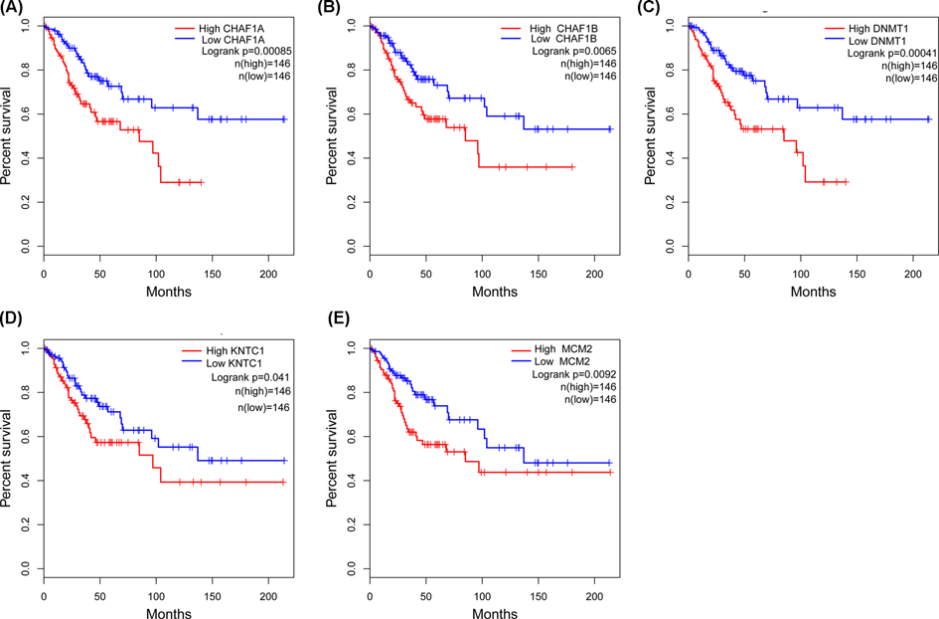
Enrichment analysis of GO and KEGG pathway of DEGs in CC (**A**) GO enrichment analysis (**B**) KEGG enrichment analysis.

GO analysis showed that DEGs were most enriched in cell cycle, chromosome, specific DNA binding, and single-stranded DNA binding. GO functional enrichment of DEGs with a *P*-value <0.05 was considered statistically significant and the results are presented in Figure 2A. KEGG pathway analysis showed that integrated DEGs were mainly enriched in four pathways consisted of cell cycle, DNA replication, p53 signaling pathway and mismatch repair (Figure 2B).

### Identification of the optimal diagnostic mRNA biomarkers for CC

**Figure 3:**
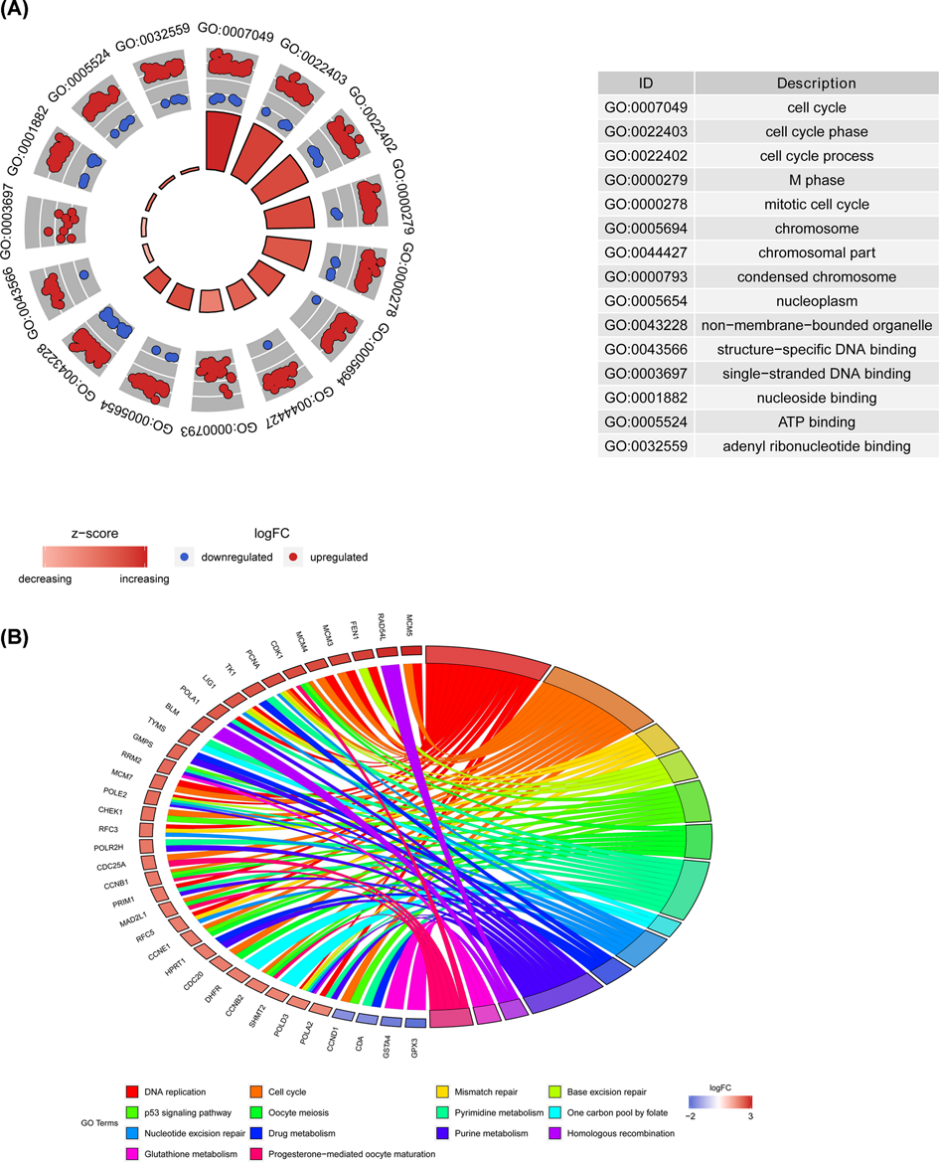
Identification of mRNA biomarkers for CC (**A**) Importance value of each DEGs ranked according to the mean decrease in accuracy by using the random forest analysis. (**B**) Variance rate of classification performance when increasing numbers of the predictive DEGs.

Based on the reduced dimension of the data, comparing CC and normal tissues identified 20 DEGs using LASSO algorithm analysis (Table 2). The random forest analysis was used to rank the 20 DEGs, according to the decrease in mean accuracy (Figure 3A). A ten-fold cross-validation result demonstrated that the average accuracy rate of ten DEGs, including DNMT1, CHAF1B, CHAF1A, MCM2, CDKN2A, KNTC1, CRISP2, KRT32, SLC5A1 and CRNN exhibited the highest score (Figure 3B). Therefore, these ten DEGs were selected as the potential optimal diagnostic mRNA biomarkers for CC and were used to establish the random forests, decision tree and SVM models.

**Table 2:** Twenty mRNAs screened by LASSO

ID	Symbol	Combined.ES	P.Value	FDR	UpDown
1029	CDKN2A	3.491349	0	0	Up
1786	DNMT1	2.594474	0	0	Up
2263	FGFR2	−1.54865	0	0	Down
3882	KRT32	−1.73084	0	0	Down
4171	MCM2	3.044853	0	0	Up
6523	SLC5A1	−2.23921	0	0	Down
6691	SPINK2	−1.80647	0	0	Down
7180	CRISP2	−1.97518	0	0	Down
8208	CHAF1B	2.189046	0	0	Up
8424	BBOX1	−2.53337	0	0	Down
9735	KNTC1	3.197516	0	0	Up
9796	PHYHIP	−2.30627	0	0	Down
10036	CHAF1A	2.314881	0	0	Up
10321	CRISP3	−3.04948	0	0	Down
10947	AP3M2	1.72574	0	0	Up
23225	NUP210	2.369988	0	0	Up
49860	CRNN	−3.22716	0	0	Down
64786	TBC1D15	1.697909	0	0	Up
65982	ZSCAN18	−2.0272	0	0	Down
79875	THSD4	−2.47196	0	0	Down

**Figure 4:**
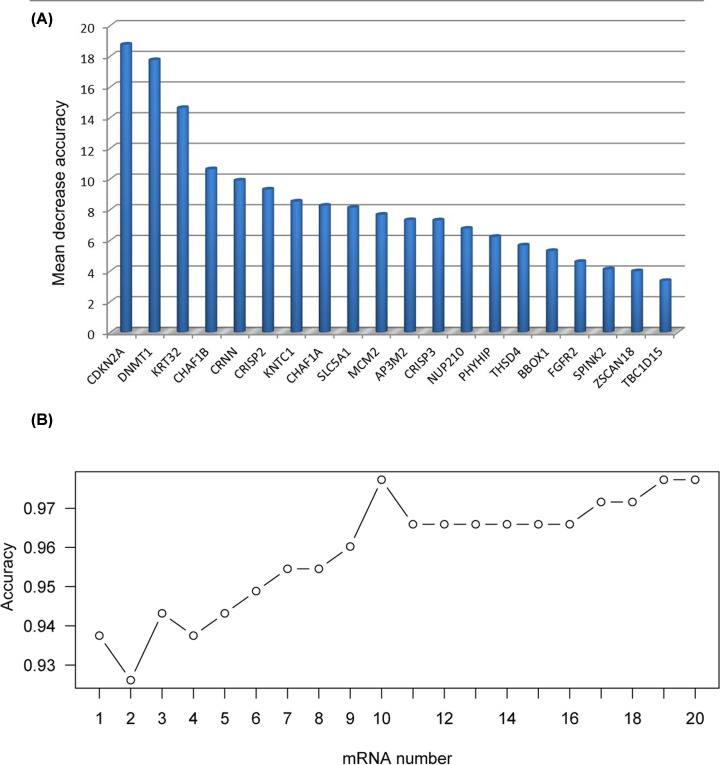
ROC analysis of five CC-specific mRNA biomarkers The ROC results of these five diagnostic mRNA biomarkers (DNMT1, CHAF1B, CHAF1A, MCM2 and CDKN2A) based on (**A**) support random forest, (**B**) decision tree model and (**C**) support vector machine model.

**Figure 5:**
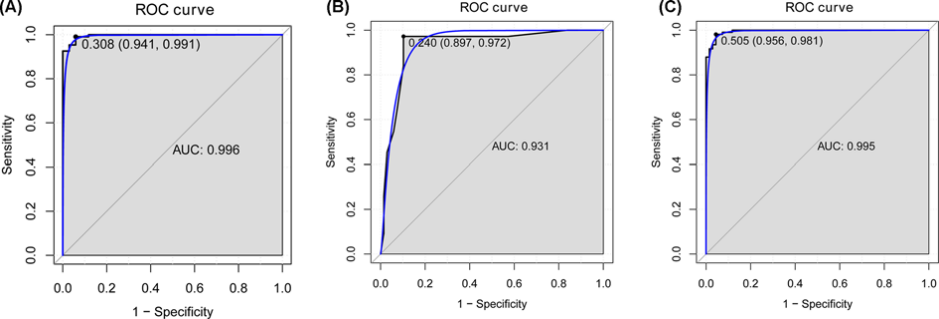
ROC curves of DNMT1, CHAF1B, CHAF1A, MCM2, KNTC1, CRISP2, CRNN, KRT32, SLC5A1 and CDKN2A (**A**) CHAF1A, (**B**) CHAF1B, (**C**) DNMT1, (**D**) CRISP2, (**E**) KRT32. (**F**) MCM2, (**G**) KNTC1, (**H**) CDKN2A, (**I**) CRNN, (**J**) SLC5A1.

The area under the ROC curve (AUC) of the random forests model was 0.996 and the specificity and sensitivity of this model were 0.941 and 0.991, respectively (Figure 4A). The AUC of the decision tree model was 0.931 and the specificity and sensitivity of this model were 0.897 and 0.972, respectively (Figure 4B). The AUC of the SVM model was 0.995, and the specificity and sensitivity of this model were 0.956 and 0.981 (Figure 4C). The AUC curves of these ten genes were shown in Figure 5, indicating strong diagnostic ability for CC.

### Identification of five DEGs associated with OS and establishment of the four-gene prognostic signature

**Figure 6:**
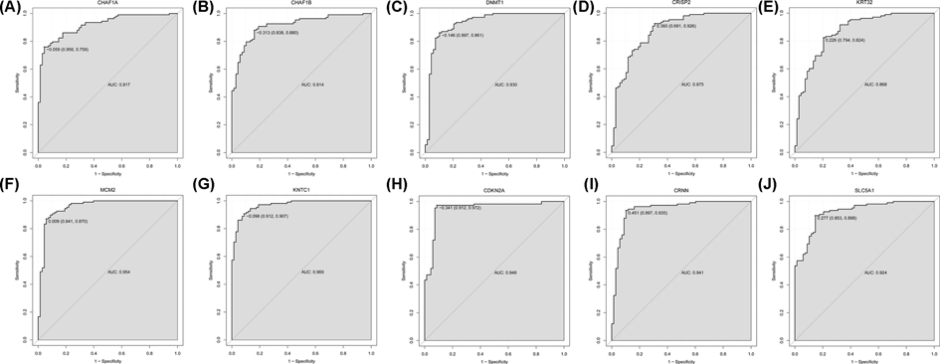
OS validation of CC patients grouped by median cutoffs of DNMT1, CHAF1B, CHAF1A, MCM2 and KNTC1 (**A**) CHAF1A, (**B**) CHAF1B, (**C**) DNMT1, (**D**) KNTC1, (**E**) MCM2.

**Figure 7:**
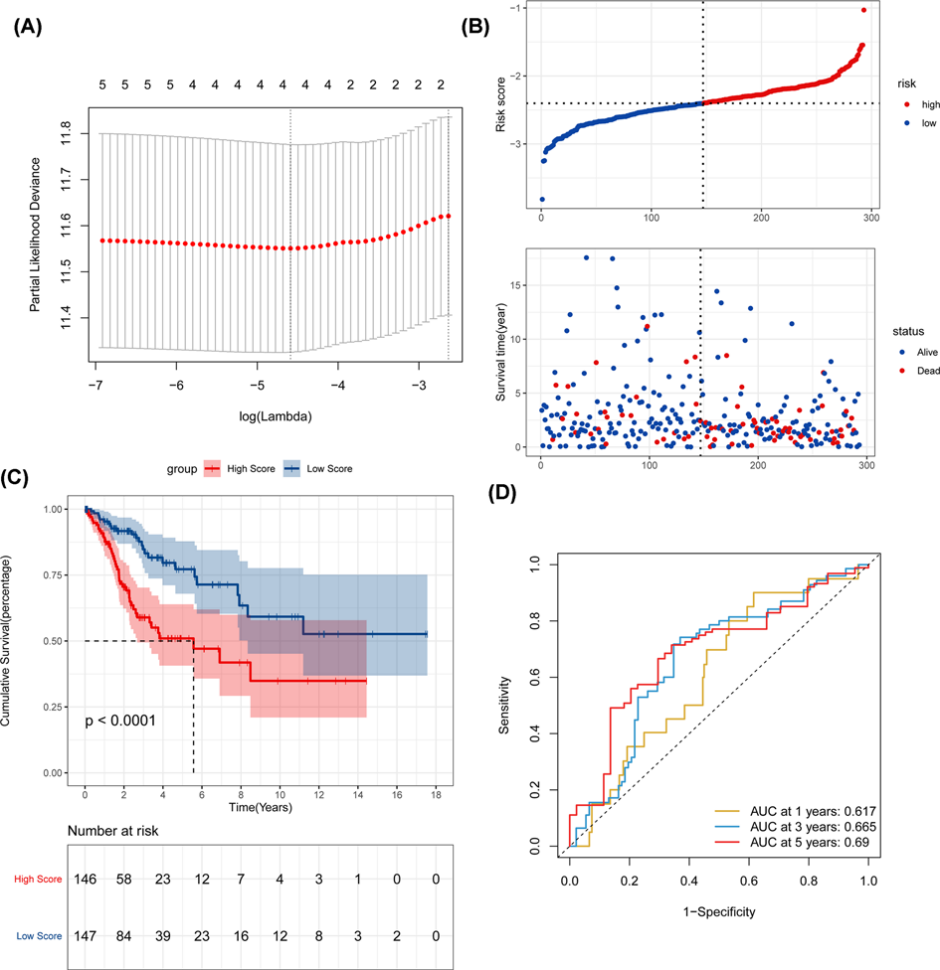
Time-dependent ROC analysis, risk score analysis, and KM analysis for the four-gene signature in CC (**A**) LASSO Cox analysis, (**B**) risk score, (**C**) KM curve of the four-gene signature. (**D**) Time-dependent ROC analysis.

In Figure 6, to identify the mRNAs which would be potentially associated with OS of CC patients, we evaluated the association between mRNAs expression and patients’ survival using KM curve and Log-rank test. The results showed that these five mRNAs (DNMT1, CHAF1B, CHAF1A, KNTC1, MCM2) were negatively correlated with OS. Five genes were subsequently used to construct a prognostic gene-signature. Finally, four-gene signature prognostic model was established. Time-dependent ROC and KM curve were used to assess the prognostic capacity of the four-gene signature. The AUCs for 1-, 3-, and 5-year OS were 0.617, 0.665, 0.690. Patients in the high-risk group showed significantly poorer OS than patients in the low-risk group (all *P*<0.001) (Figure 7A–D).

### Correlation between candidate genes and clinical features in CC patients

Downloading the TCGA clinical data in LinkedOmics online tool, we analyzed the relationship between selected genes and clinical features in CC patients. The CHAF1B and KNTC1 in CC patients were significantly correlated with tumor purity. The CHAF1B was also significantly correlated with radiation therapy and MCM2 was significantly correlated with histological type (Table 3).

**Table 3:** Relationship between selected candidate genes and clinical features in CC

Item	*n*	DNMT1		CHAF1B		CHAF1A		MCM2		KNTC1	
		Statistic	*P*-value	Statistic	*P*-value	Statistic	*P*-value	Statistic	*P*-value	Statistic	*P*-value
years_to_birth (Spearman Correlation)	307	−0.02	0.72	0.04	0.56	0.00	0.98	0.05	0.45	0.01	0.90
Tumor_purity (Spearman Correlation)	307	0.05	0.37	0.22	0.00	0.04	0.55	0.10	0.10	0.19	0.00
ethnicity (Wilcox Test)	Hispanic or Latino 24	−0.01	0.12	−0.03	0.16	−0.01	0.56	−0.01	0.37	−0.02	0.42
	not Hispanic or Latino 171										
race (Kruskal–Wallis Test)	Asian 20	2.05	0.73	7.74	0.10	1.97	0.74	7.92	0.09	4.21	0.38
	White 211										
	Black or African American 30										
radiation_therapy (Wilcox Test)	Yes 129	−0.01	0.37	−0.02	0.04	−0.03	0.10	−0.02	0.19	−0.01	0.18
	NO 55										
pathology_T_stage (Kruskal–Wallis Test)	T1 141	4.04	0.40	2.43	0.66	2.17	0.70	0.90	0.93	1.68	0.79
	T2 72										
	T3 21										
	T4 10										
pathology_N_stage (Wilcox Test)	N0 135	-0.01	0.41	−0.02	0.37	0.02	0.72	0.00	0.28	−0.01	0.82
	N1 60										
pathology_M_stage (Wilcox Test)	M0 116	0.00	0.30	−0.02	0.41	−0.05	0.07	−0.03	0.74	−0.01	0.99
	M1 10										
histological_type (Kruskal–Wallis Test)	Cervical squamous cell carcinoma 254	1.68	0.89	5.32	0.38	11.32	0.05	26.49	0.00	3.52	0.62
	Endocervical type of adenocarcinoma 21										

### Quantitative RT-PCR confirmation and validation in datasets

**Figure 8:**
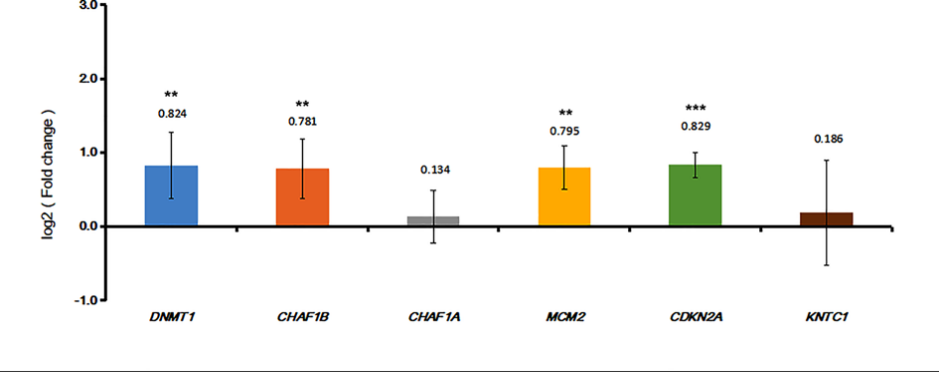
qRT-PCR results of five DEGs (DNMT1, CHAF1B, CHAF1A, MCM2, KNTC1 and CDKN2A) in CC *P<0.05, **P<0.01, ***P<0.001.

To indicate the results of integrated analysis, we selected five mRNAs (DNMT1, CHAF1B, CHAF1A, MCM2, KNTC1 and CDKN2A) in CC versus normal control. Based on the results of quantitative RT-PCR, the expression of DNMT1, CHAF1B, CHAF1A, MCM2, KNTC1 and CDKN2A were all up-regulated, which were consistent with the results of our integrated analysis (Figure 8).

**Figure 9:**
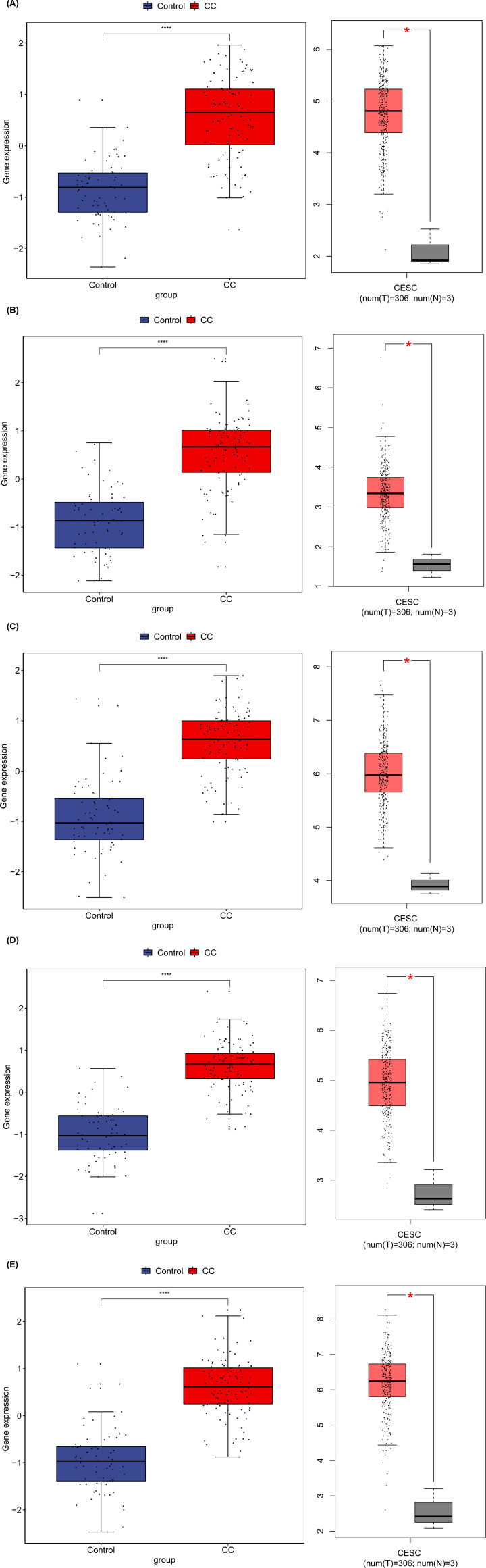
Validation of the expression of candidate genes in CC tissues and normal tissues in GEO and TCGA (**A**) CHAF1A, (**B**) CHAF1B, (**C**) DNMT1, (**D**) KNTC1, (**E**) MCM2. *P<0.05, **P<0.01, ***P<0.001.

**Figure 10:**
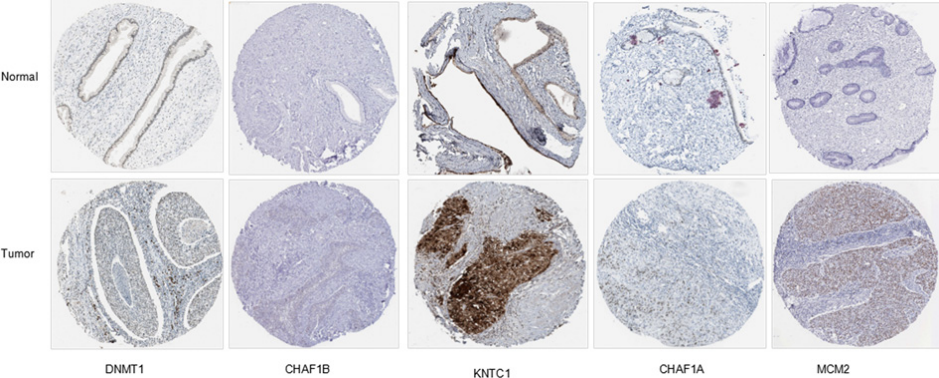
Immunohistochemical staining of candidate genes in CC tissues and normal tissues in the HPA database

Using the GEO and TCGA data, we analyzed the expression of the five selected up-regulated genes in CC tissues and normal tissues. The results showed that the DNMT1, CHAF1B, CHAF1A, MCM2 and KNTC1 were highly expressed in CC tissues, and the differences were statistically significant (Figure 9). The immunohistochemical staining is displayed in Figure 10.

## Discussion

CC is one of the most general malignant tumors in gynecology, and it is also one of the main causes of female cancer deaths [15]. Statistically, 80 percent of patients develop aggressive cancer once diagnosed and the age of diagnosed patients is tardily decreasing [16]. Furthermore, on account of metastasis and recurrence, the incidence and mortality of CC remain high [17]. At the same time, reliable and specific biomarkers for the diagnosis and prognosis of CC are scarce and lack exploration. Thus, it is urgent to find diagnostic biomarkers and new therapeutic targets to predict the survival of CC. Gene expression microarrays have been broadly used in the study of genes related to tumors, offering broad prospects for molecular therapy of drugs [18]. A battery of biomarkers have been suggested as potential targets for the diagnosis and prognosis of CC [19,20].

DNMT1 is a member of the DNA methyltransferase family, responsible for the DNA methylation of cytosine-phosphoguanine (CpG) island upstream of tumor suppressor genes [21,22]. DNMT1 is the most important one. Abnormal expression of DNMT1 can lead to abnormal methylation of some tumor suppressor genes CpG islands, which in turn leads to inactivation of tumor suppressor genes and cell carcinogenesis [23]. Highly expressed DNMT1 is not only detected in a variety of tumor cells, but also appears before DNA methylation [24]. Previous studies have shown that DNMT1 is an essential substance for the maintenance of cancer stem cells (CSCs) in various cancers such as prostate cancer, pancreatic cancer and breast cancer [25–27]. For example, DNMT1 induces histone demethylation of H3K9me3 and H3K27me3 on the promoters of Zeb2 and KLF4 in prostate cancer cells [26]. More importantly, a recent study showed that DNMT1 is up-regulated in breast tumors. DNMT1 inhibition or DNMT1 induced Islet-1 (ISL1) hypermethylation/down-regulation limits the number of CSCs in breast cancer cells [25]. In our study, DNMT1 was up-regulated in CC versus normal control, and may be as an oncogene in development of CC. Furthermore, DNMT1 was significantly associated with OS time and one of the diagnostic markers. Thus, we speculated that the expression level of DNMT1 has both diagnostic and prognostic values.

MCM2 is a component of the DNA replication licensing complex (MCM2-7) that has been found to mainly localize to the nucleus in eukaryotic cells. MCM2 has been recognized as a useful marker in screening for cervical carcinoma oral squamous cell carcinoma and medulloblastoma. Overexpression of MCM2 occurs frequently in CC, especially in cases with persistent high-risk HPV infection [28]. Many studies mainly focus on the analysis of biomarkers related to precancerous lesions, but only a few studies have confirmed the prognostic influence of MCM2 expression in the progression of aggressive CC [29,30]. Wang et al. found that MCM2 was a prognostic biomarker in CC [31]. We also observed high expression levels of MCM2 in CC tissues. Aihemaiti et al. reported that cytoplasmic rather than nuclear accumulation of MCM2 is related to improved survival for patients with ovarian clear cell carcinoma, which maybe associated with MCM2-mediated DNA damage-induced apoptosis [32]. It was therefore hypothesized in the present study that MCM2 may serve an important role in CC. In addition, MCM2 was also correlated with diagnosis and prognosis for CC.

CHAF1A which is also called P150 is the subunit of Chromatin Assembly Factor-1 (CAF-1) [33–37]. CHAF1A enhances Gfi1-mediated transcriptional repression and occupies Gfi1 target gene promoters in transfected cells [38]. Recently, CHAF1A has been associated with the development and progression of solid tumors, including breast cancer, prostate squamous cell carcinoma, hepatocellular carcinoma (HCC), glioma and neuroblastoma [39–43]. A number of studies have shown that CHAF1A is also highly expressed in breast cancer, colon cancer, CC and other tumors, and can be used as a potential marker for judging the prognosis of tumor patients and a target for tumor treatment [44]. However, the expression and biological function of CHAF1A in CC remains largely unidentified.

Chromatin assembly factor 1, subunit B (CHAF1B), the p60 subunit of CAF-1, plays a vital role in DNA replication and chromatin assembly in proliferating tissues [45]. Tumor cells are usually characterized by a high proliferation rate. Therefore, it is speculated that CHAF1B plays an important role in the pathogenesis of malignant tumors. Researches have shown that up-regulated CHAF1B is significantly correlated with poor outcomes and that CHAF1B has potential in predicting the prognosis in several cancers, including CC [46–48]. These reported results were consistent with our study.

KNTC1 gene is distributed in the cytoplasm, nucleus, chromosomal centromere, centromere, cytoskeleton and in the spindle fiber. KNTC1 has been studied in a variety of human malignancies and is related to the pathological grade of tumor tissues [49]. As previously described, the expression of KNTC1 in three esophageal squamous cell carcinoma (ESCC) cell lines and established that all the tested cell lines showed that it is positively expressed [50]. Previous studies have shown that the KNTC1 transcriptional activity changes with the size of tumor in patients with oral squamous cell carcinoma [51]. Also, compared with the healthy control group, the KNTC1 expression in neuroblastoma samples increased statistically. Several bioinformatics studies have also established that the KNTC1 gene could be one of the vital genes associated with cancer development, including HCC, Pancreatic cancer (PC), and nasopharyngeal carcinoma (NPC) [52]. Chen et al. reported that KNTC1 gene is closely related to the poor prognosis of CC, which was consistent with our findings [49].

In conclusion, we obtained several DEGs in CC and found that overexpression of DNMT1, CHAF1B, CHAF1A, MCM2, KNTC1 in tumor tissues predicted poor survival in CC. These DEGs also have diagnostic value for CC at early stage. We hypothesized that DNMT1, CHAF1B, CHAF1A, MCM2 and KNTC1 may be potential therapeutic targets for CC. We analyzed these genes at the transcriptional and protein levels, validated by qRT-PCR and immunohistochemical staining.

## Data Availability

The datasets used and/or analyzed during the current study are available from the corresponding authors on reasonable request.

## Abbreviations

AUC: area under the ROC curve
CAF-1: chromatin assembly factor-1
CC: cervical cancer
CHAF1A: chromatin assembly factor 1, subunit A
CHAF1B: chromatin assembly factor 1, subunit B
CpG: cytosine-phosphoguanine
CSC: cancer stem cell
DEG: differentially expressed gene
DNMT1: DNA methyltransferase 1
FDR: false discovery rate
GEO: Gene Expression Omnibus
GEPIA: Gene Expression Profiling Interactive Analysis
GO: gene ontology
HCC: hepatocellular carcinoma
HPV: Human papillomavirus
KEGG: Kyoto Encyclopedia of Genes and Genomes
KM: Kaplan–Meier
KNTC1: kinetochore-related protein 1
OS: overall survival
ROC: Receiver operating characteristic
SCC: squamous cell carcinoma
SVM: Support Vector Machine
Zeb2: Zinc Finger E-Box Binding Homeobox 2
H3K27me3: tri-methylation at lysine 27 of histone H3
H3K9me3: tri-methylation of lysine 9 on histone H3
