# Correction: Five candidate biomarkers associated with the diagnosis and prognosis of cervical cancer

**DOI:** 10.1042/BSR-20204394_COR

**Published:** 2021-10-04

**Keywords:** cervical cancer, differentially expressed genes, machine learning

The authors of the original article “Five candidate biomarkers associated with the diagnosis and prognosis of cervical cancer” (*Biosci Rep* (2021) **41**(3), https://doi.org/10.1042/BSR20204394) would like to correct Figure 6, due to issues with the labelling of the red and blue lines within the graphs. The authors declare that this adjustment does not change the results or conclusions of their paper, and express their apologies for any inconvenience that this error has caused to the readers. The corrected version of Figure 6 is presented here.

**Figure 6:**
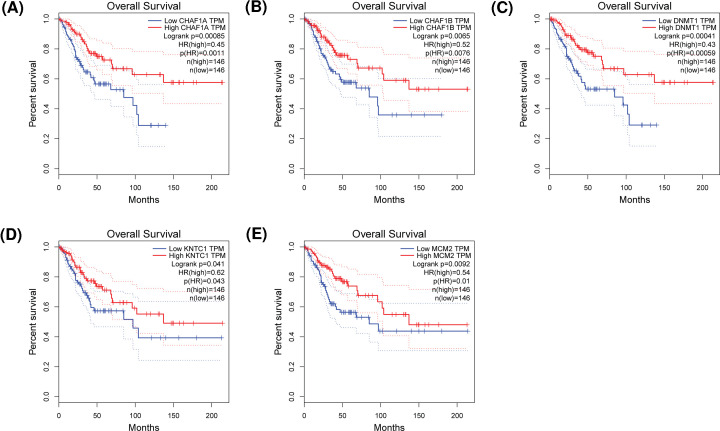
OS validation of CC patients grouped by median cutoffs of DNMT1, CHAF1B, CHAF1A, MCM2 and KNTC1 (**A**) CHAF1A, (**B**) CHAF1B, (**C**) DNMT1, (**D**) KNTC1, (**E**) MCM2.

